# Development of a human-computer collaborative sleep scoring system for polysomnography recordings

**DOI:** 10.1371/journal.pone.0218948

**Published:** 2019-07-10

**Authors:** Sheng-Fu Liang, Yu-Hsuan Shih, Peng-Yu Chen, Chih-En Kuo

**Affiliations:** 1 Department of Computer Science and Information Engineering, National Cheng Kung University, Tainan, Taiwan; 2 AI Biomedical Research Center at NCKU, Ministry of Science and Technology, Tainan, Taiwan; 3 Department of Automatic Control Engineering, Feng Chia University, Taichung, Taiwan; Zapadoceska univerzita, CZECH REPUBLIC

## Abstract

The overnight polysomnographic (PSG) recordings of patients were scored by an expert to diagnose sleep disorders. Visual sleep scoring is a time-consuming and subjective process. Automatic sleep staging methods can help; however, the mechanism and reliability of these methods are not fully understood. Therefore, experts often need to rescore the recordings to obtain reliable results. Here, we propose a human-computer collaborative sleep scoring system. It is a rule-based automatic sleep scoring method that follows the American Academy of Sleep Medicine (AASM) guidelines to perform an initial scoring. Then, the reliability level of each epoch is analyzed based on physiological patterns during sleep and the characteristics of various stage changes.

Finally, experts would only need to rescore epochs with a low-reliability level. The experimental results show that the average agreement rate between our system and fully manual scorings can reach 90.42% with a kappa coefficient of 0.85. Over 50% of the manual scoring time can be reduced. Due to the demonstrated robustness and applicability, the proposed approach can be integrated with various PSG systems or automatic sleep scoring methods for sleep monitoring in clinical or homecare applications in the future.

## Introduction

Sleep disorders, such as insomnia and obstructive sleep apnea can seriously affect the quality of life of patients. The prevalence of insomnia symptoms, without using restrictive criteria, is approximately 33% of the general population [1]. The diagnosis of sleep disorders involves overnight polysomnographic (PSG) recordings of a sleeping patient, including electroencephalograms (EEG), electrooculograms (EOG) and electromyograms (EMG). The PSG recordings are scored by a well-trained expert according to the Rechtschaffen & Kales (R&K) rules or the American Academy of Sleep Medicine (AASM) guidelines [2] to classify each epoch (i.e., 30-s data) into one of the sleep stages, including wakefulness (stage W), non-rapid eye movement (stages N1, N2, and N3) and rapid eye movement (stage R).

Because visual sleep scoring is a time-consuming and subjective process, automatic sleep staging methods based on multi-channel signals, including EEG, EOG, and EMG were developed [3] [4–8]. Scoring methods based on single-channel EEG or EOG were also developed for home based sleep-monitoring [9–18]. Recently, deep learning, a branch of machine learning that utilizes the structure of Deep Belief Nets (DBNs), Convolutional Neural Networks (CNNs), or Long Short-Term Memory (LSTM) to learn hierarchical representations or features from input data, has also been employed in sleep staging [19–26]. The AASM interscorer reliability program [27] reported that interscorer agreement is 82.6%. Although the reported overall agreement from some automatic sleep scoring methods can reach higher than 82.6%, experts often manually rescore the PSG data because the methods behind the scoring process are poorly understood. Instead of fully automatic sleep scoring, some sleep centers use automatic methods to oversee some of the decisions [28]. Nevertheless, the experts must always manually rescore the PSG data. In other words, these automatic scoring methods cannot cooperate with the scorer to effectively reduce the amount of time and effort spent scoring.

According to the AASM interscorer program report, agreement are reduced during stage transitions, such as from stage W to N1, stage N2 to N3, and stage N2 to R [27]. Even the experts with three or more years of sleep scoring experience, the agreement reduces 10.5% during the stage transitions from W to N1 compared with the average agreement corresponding to N1. Similarly, the agreements reduce 17.5% and 35% during N1 to N2 and N2 to N3 transitions compared with the average agreements corresponding to N2 and N3, respectively. M. Younes et al. also reported large disagreements between transition from N1 to N2 and N2 to N3 [29]. Interscorer variability is mostly due to epochs that are difficult to identify. The availability of digitally identified events (e.g., spindles) or calculated variables (e.g., depth of sleep, delta wave duration) during scoring may greatly reduce scoring variability.

Dimitriadis SI et al. proposed a single-EEG-sensor automatic sleep stage classification technique based on the dynamic reconfiguration of different aspects of cross-frequency coupling [30]. A high classification performance was achieved (accuracy was 94.4% across 20 folds), the recordings for evaluating classification performance were taken from 20 healthy young adults. The results can be replicated in an external dataset of 77 subjects. Aboalayon K. et al. proposed a novel and efficient technique to identify sleep stages using new statistical features applied to the single-channel EEG signal [31]. They used the same dataset in Dimitriadis et al. [30], but less data were included. Zhang et al. proposed an automatic sleep stage method that combined a sparse deep belief net with multiple classifiers [32]. However, again the data used for developing the system included only ten subjects (eight of which did not have sleep disorders). As the study in [30], the data from a larger patient pool and evaluation with multiple datasets is the trend for the development of automatic sleep scoring methods, how to cooperate with the scorer is the next challenge for clinical applicability.

Taking into account the above limitations, we developed a sleep scoring system that experts would be willing to use, a human-computer collaborative sleep scoring (HCSS) system. The goals of the HCSS system are as follows: 1) to provide a reliability score for each result, so experts can assess the automatic scoring method; 2) to reduce manual scoring time, where experts only need to rescore epochs with a low reliability score; and 3) to ensure the final scoring results reliable and accepted by experts. To achieve these goals, a rule-based automatic sleep scoring method [5] following AASM guidelines was utilized as the foundation of the system to perform the initial scoring. Next, a reliability analysis strategy was developed to compare the sleep features, stage change distance, and stage change frequency of the initial scoring results. Finally, experts rescored epochs with low levels of reliability to fine-tune the results. The overnight PSG recordings from 30 subjects were used in the experiment to evaluate the performance of the proposed system including the agreement between manual scorings and the proposed HCSS system. The reduction in time spent manually scoring through use of the HCSS system is also reported.

## Materials and methods

### Subjects and recordings

The experimental procedures in this paper were reviewed and approved by the Institutional Review Board of Human Experiment and Ethics Committee, National Cheng Kung University Hospital, Taiwan. Overnight polysomnographic (PSG) sleep recordings from 30 subjects (17 males and 13 females) ranging from 18 to 24 years in age were utilized in the experiments. The recordings included 2 EEG channels (C3-M2 and C4-M1), according to the international 10–20 standard system, two EOG channels (the above right and lower left outer canthus), and a chin EMG channel which was acquired through a Siesta 802 PSG (Compumedics, Inc.). The sample rate was 256 Hz with a 16-bit resolution. The filter settings of the cut-off frequencies were 0.5–30 Hz for EEG/EOG and 5–100 Hz for EMG. All PSG recordings were scored by a sleep expert (gold standard) according to the AASM rules with a 30-s epoch; the scoring results were utilized as the gold standard. The manually scored epochs were each classified into one of the sleep stages, stage W, stage N1, stage N2, stage N3, and stage R. The range of the subjects’ sleep efficiency (SE), the ratio of time spent asleep (total sleep time) to the amount of time spent in bed, ranged from 56% to 97%. Subjects with good (N = 15) and poor (N = 15) SE were included to evaluate the robustness and reliability of our system. Two scorers (who have over 800 hours of experience in PSG recording and scoring), scorers 1 and 2, were invited to participate in the experiments for performance evaluation.

### System

**Fig 1:**
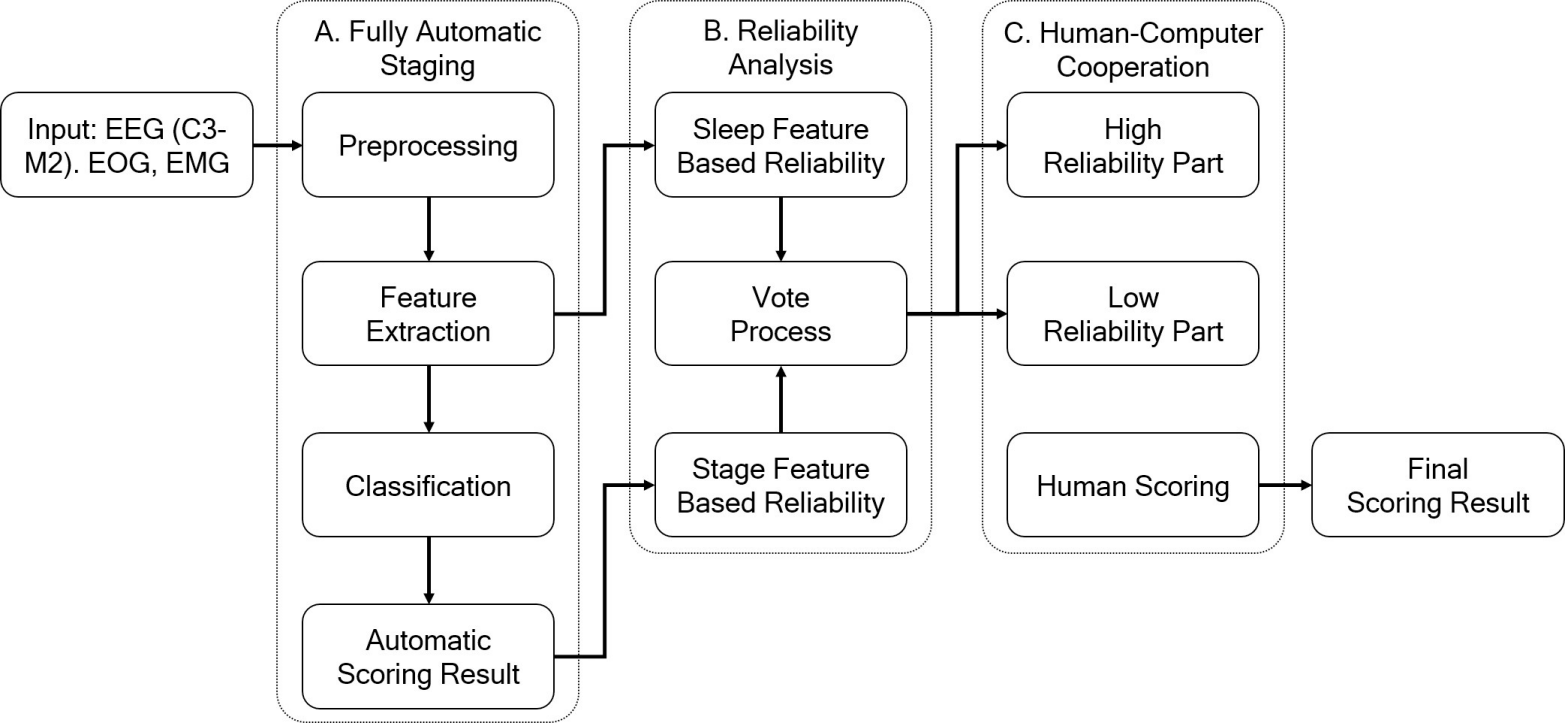
The flow chart of the human-computer collaborative sleep scoring system. The system consists of (A) fully automatic scoring, (B) reliability analysis, and (C) human-computer collaboration.

Fig 1 shows a flow chart of the human-computer collaborative sleep scoring system. The system includes three parts: (A) fully automatic scoring, (B) reliability analysis, and (C) human-computer collaboration. Details of the scoring system are described below.

### Fully automatic scoring

In this study, for the initial step, we employed a rule-based sleep scoring method [5, 33] based on a decision tree to automatically identify the sleep stages. Stages that are easier to identify were processed at nodes in the upper layers, while stages that are more difficult to distinguish were processed at nodes in the lower ones. A distribution distance measure of the feature is used to select the effective features of the nodes in the decision tree. There is a total of 13 decision nodes in the decision tree and 12 features for automatic sleep scoring. After classifying the sleep stage using the decision tree, a smoothing process, that considers the temporal context information, was applied for continuity. These rules refer to the relationship between epochs before and after the current epoch.

**Fig 2:**
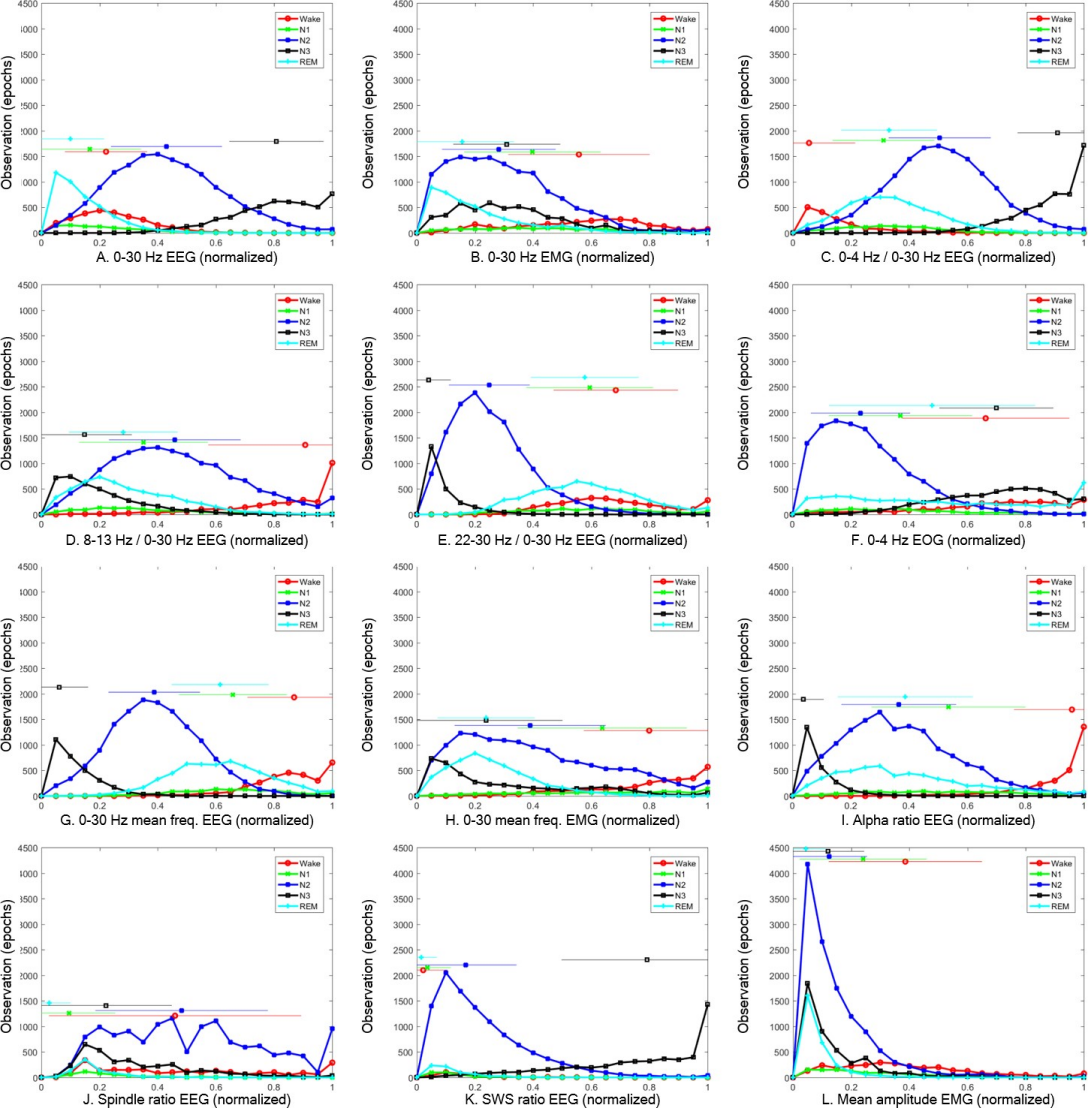
Histogram of 12 sleep features in Wake, N1, N2, N3, and REM stages of 30 PSG data. The X-axis represents the normalized feature values and the Y-axis represents the number of epochs. Features are power: (A) 0–30 Hz EEG, (B) 0–30 Hz EMG; power ratio: (C) 0–4 Hz / 0–30 Hz EEG, (D) 8–13 Hz / 0–30 Hz EEG, (E) 22–30 Hz / 0–30 Hz EEG; power (F) 0–4 Hz EOG; spectral frequency: (G) 0–30 Hz mean frequency EEG, (H) 0–30 Hz mean frequency EMG; duration ratio: (I) alpha ratio EEG, (J) Spindle ratio EEG, (K) SWS ratio EEG; amplitude: (L) mean amplitude EMG.

Table 1 shows the relationship between the features and the R&K rules. The feature “Alpha E” correspond to the duration of the epoch and consists of alpha (8–13 Hz) activity for stage Wake. The “Spindle E” feature corresponds to the duration of spindle activity for stage S2. The “0–4 E” and “SWS E” features correspond to the magnitude and duration of delta activity for stage SWS. The “Amp M” feature corresponds to the magnitude of EMG for stage REM. Fig 2 shows a histogram of the 12 sleep features in Wake, N1, N2, N3, and REM stages from 30 PSG recordings.

**Table 1:** Features for automatic sleep scoring.

No.	Type	Feature	Source	Label
1	PS	Total power of 0–30 Hz	EEG	0–30 E
2	PS	Total power of 0–30 Hz	EMG	0–30 M
3	PR	0–4 Hz/0–30 Hz	EEG	0–4 E
4	PR	8–13 Hz/0–30 Hz	EEG	8–13 E
5	PR	22–30 Hz/0–30 Hz	EEG	22–30 E
6	PR	0–4 Hz/0–30 Hz	EOG	0–4 O
7	SF	Mean frequency of 0–30 Hz	EEG	Mean(fre.) E
8	SF	Mean frequency of 0–30 Hz	EMG	Mean(fre.) M
9	DR	Alpha ratio	EEG	Alpha E
10	DR	Spindle ratio	EEG	Spindle E
11	DR	SWS ratio	EEG	SWS E
12	EMG energy	Mean amplitude	EMG	Amp M

PS, power spectrum; PR, power ratio; SF, spectral frequency; DR, duration ratio.

### Reliability analysis

After the fully automatic processing of the PSG recordings, we obtained data for the 12 features used in the automatic staging as well as the initial scoring results. Three of these features and the initial scoring results were used to analyze the staging reliability of each epoch. The reliability analysis is based on the sleep features and the stage information is described as follows.

### Slow wave related (SWR) features

After analysis by the decision tree, the stage change between stages N2 and N3 had high disagreement with the AASM Interscorer Reliability Program [27]. The slow wave is the main feature in N3, and we observed that the values of the slow wave features were close to the threshold when the sleep stage changes to N3 from N2 or to N2 from N3. Therefore, we calculated the average and standard deviation (STD) of three slow wave related (SWR) features including the EEG total power of 0–30 Hz (0–30 E), slow wave ratio (SWS E), and the EOG power ratio of 0-4/0-30 Hz (0–4 O) in the N2 and N3 stages of the training data from a private database [5]. The mean values of the features in N3 are higher than those in N2, so we set the range between mean + 1*STD of N2 and mean– 1*STD of N3 for each feature as the boundary range with a high possibility of misclassification. If more than one SWR features are in the boundary range, an epoch identified as N2 or N3 stage by the automatic scoring system is annotated with a low reliability score and is flagged to be rescored by an expert.

**Fig 3:**
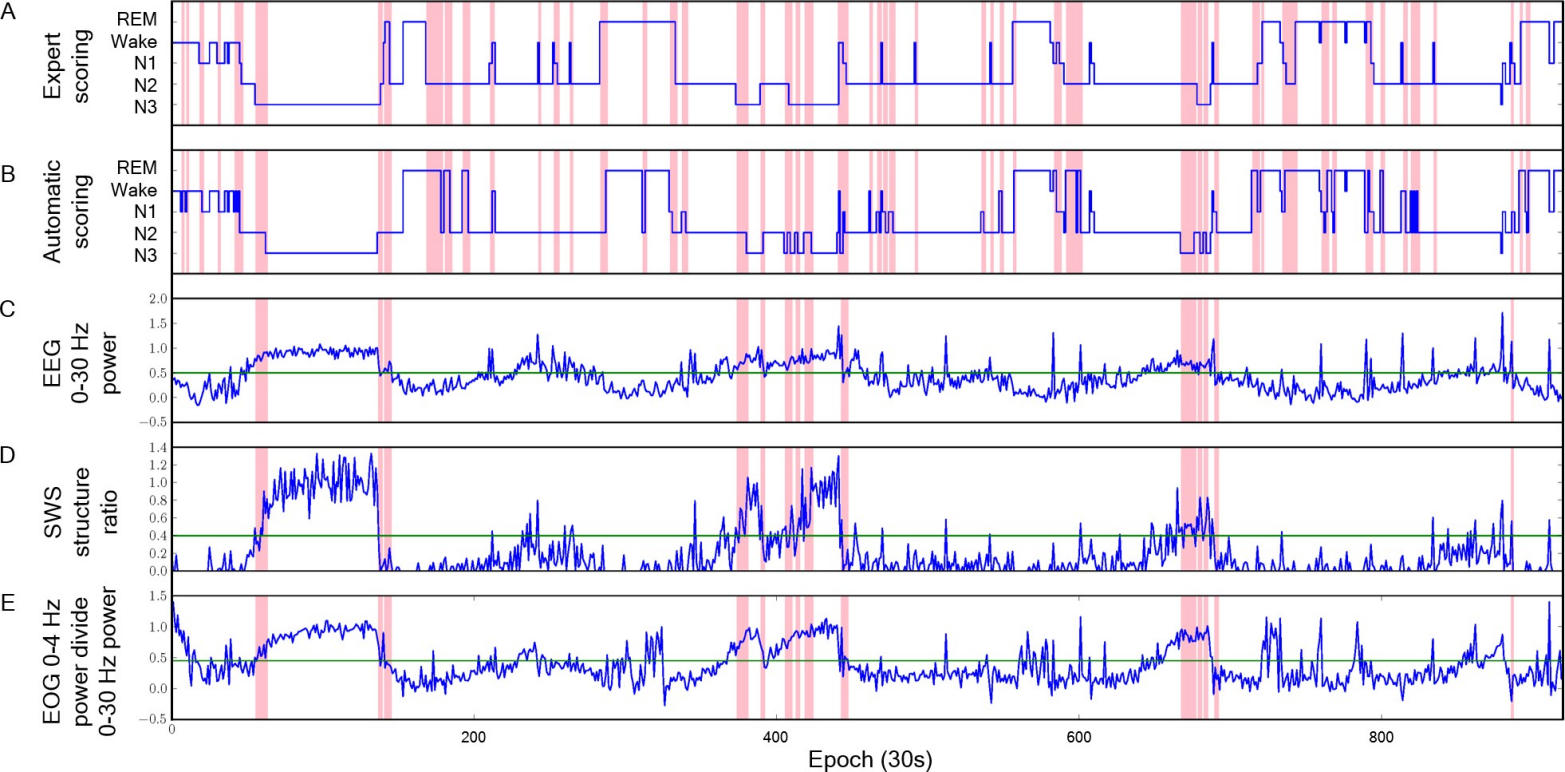
The hypnogram and SWR features of subject no. 1. The hypnograms scored by gold standard (A) and the automatic staging system (B). The values of features 0–30 E (C), SWS E (D), and 0–4 O (E).

Fig 3 illustrate the SWR features in the boundary ranges for the epochs close to the start or end of stage N3. Fig 3(A) and 3(B) are the hypnograms scored by gold standard and the automatic staging system, respectively. Fig 3(C)–3(E) show the values of features 0–30 E, SWS E, and 0–4 O, respectively. The red lines in Fig 3(A) and 3(B) indicate the epochs where the scorings results of the expert and the computer are different. The red lines in Fig 3(C)–3(E) indicate the disagreements during the start or end of stage N3.

### Stage change distance (SCD)

**Fig 4:**
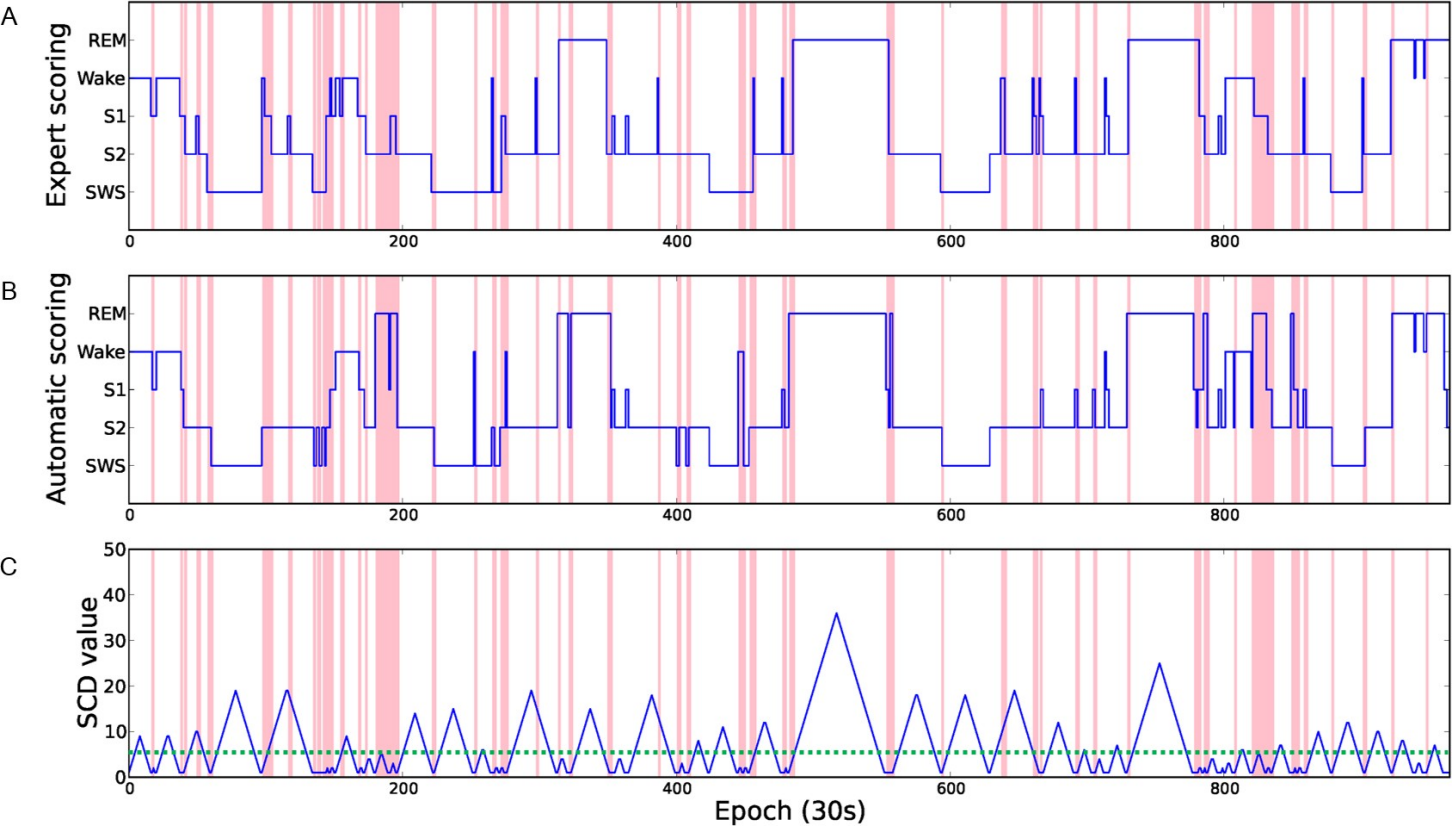
The hypnogram and SCD value of PSG from subject no. 3. The hypnograms scored by gold standard (A) and the automatic staging system (B). The value of feature SCD (C). The red lines indicate disagreement between the expert and the automatic scoring system.

The disagreements of sleep scoring mostly occurred in epochs identification during stage changes and epochs separated from the stage changes that are relatively easy to identify. Therefore, we calculated the minimum distance from the current epoch to the epoch belongings to other stages and the distance (number of epochs) was defined as stage change distance (SCD). Fig 4 shows the hypnogram and SCD values of the PSG from subject no. 3. The red lines in Fig 4 indicate the epochs where the scorings results of the expert and the computer are different. The green dash line is the threshold between high and low reliability score of SCD. It can be observed that most of the disagreements occur in epochs with a low SCD value. According to our experiments, approximate 95% of disagreements occur in the epochs whose SCD values are greater than or equal to 6. As a result, the epoch is labeled as low reliability if the SCD value is less than 6.

### Stage change frequency (SCF)

**Fig 5:**
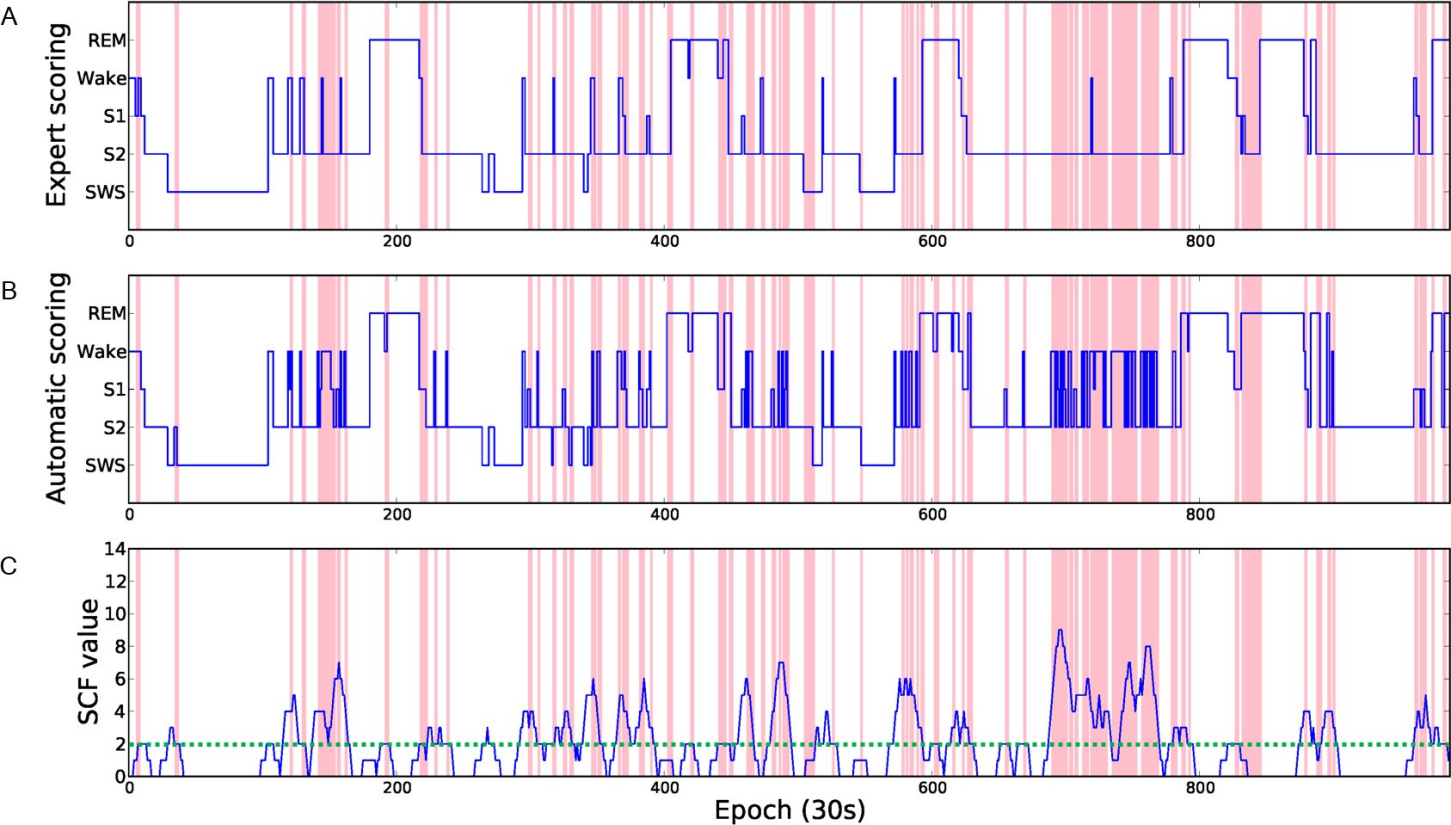
The hypnogram and SCF values of subject no. 5. The hypnograms scored by gold standard (A) and the automatic staging system (B). The value of feature SCF (C). The red lines indicate disagreement between the expert and the automatic scoring system.

When a sleep stage is sustained for a long period of time, it is relatively stable. In contrast, the periods with frequent stage changes cause high disagreement. Therefore, the number of stage changes in a given window, called the stage change frequency (SCF), was also calculated as a feature to quantify the stability of an epoch. When scoring the current epoch, an expert usually refers to the adjacent 3–4 epochs, therefore, we set 11 adjacent epochs (the current epoch, and its previous and succeeding five consecutive epochs) as the windows size to calculate a SCF value. According to statistical analysis 73% of disagreements occurred in the epochs with a SCF value above 2, and 67% of agreements occurred in the epochs with SCF values equal to or less than 2. Therefore, if the change times in the window is less than or equal to 2, the epoch is labeled as high reliability. Otherwise, the epoch is labeled as low reliability. Fig 5 shows the hypnogram and SCF values from PSG of subject no. 5, and it can be observed that most disagreements are within epochs with SCF values higher than 2 (green dashed line).

### Voting process and human-computer collaboration

**Fig 6:**
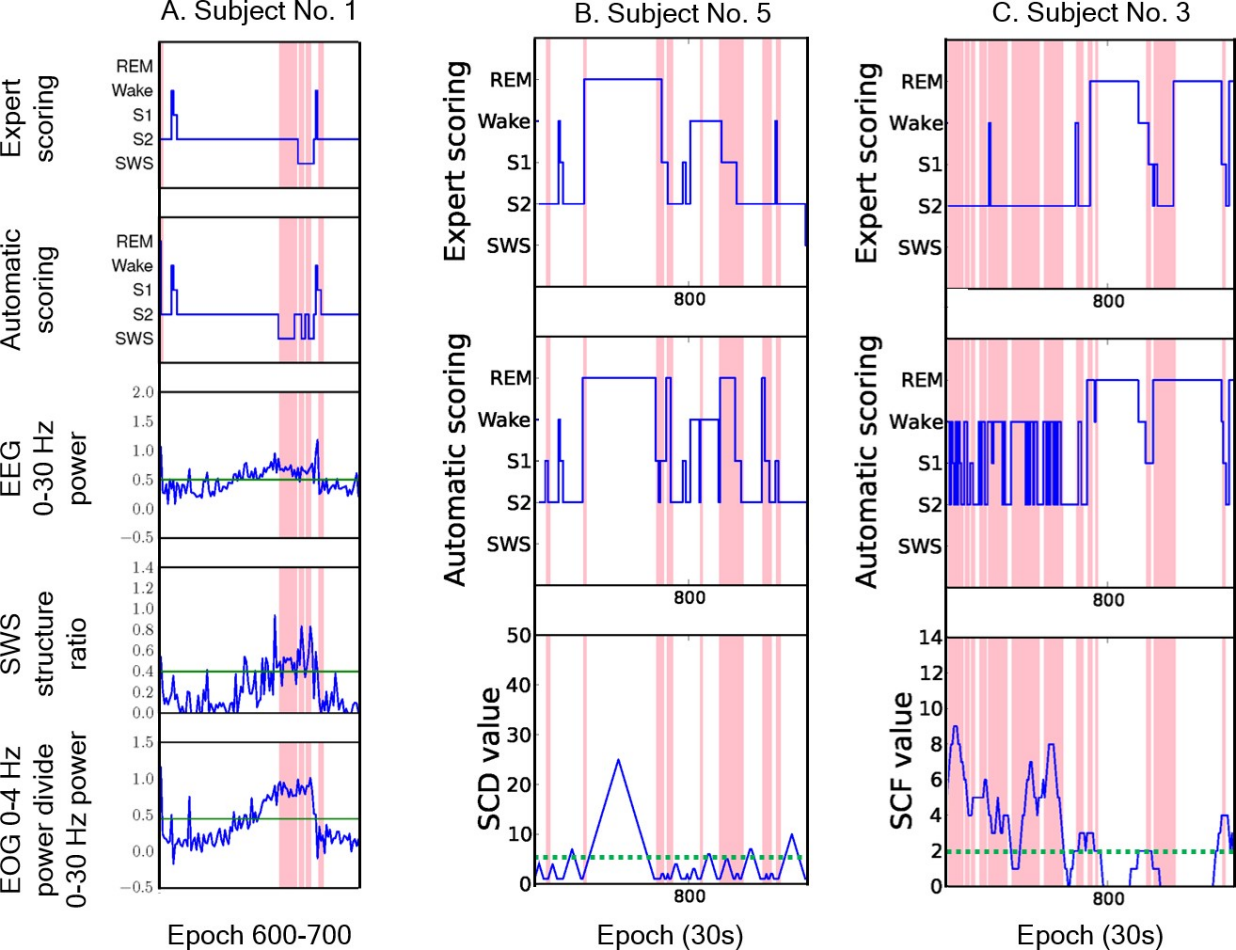
Reliability analysis examples. Disagreements can be detected by using the SWR (A), SCD (B) and SCF (C) features.

As shown in Fig 6, the majority of disagreements occur in epochs during the transition from one stage to another, which can be detected by using three features, SWR, SCD and SCF.

**Fig 7:**
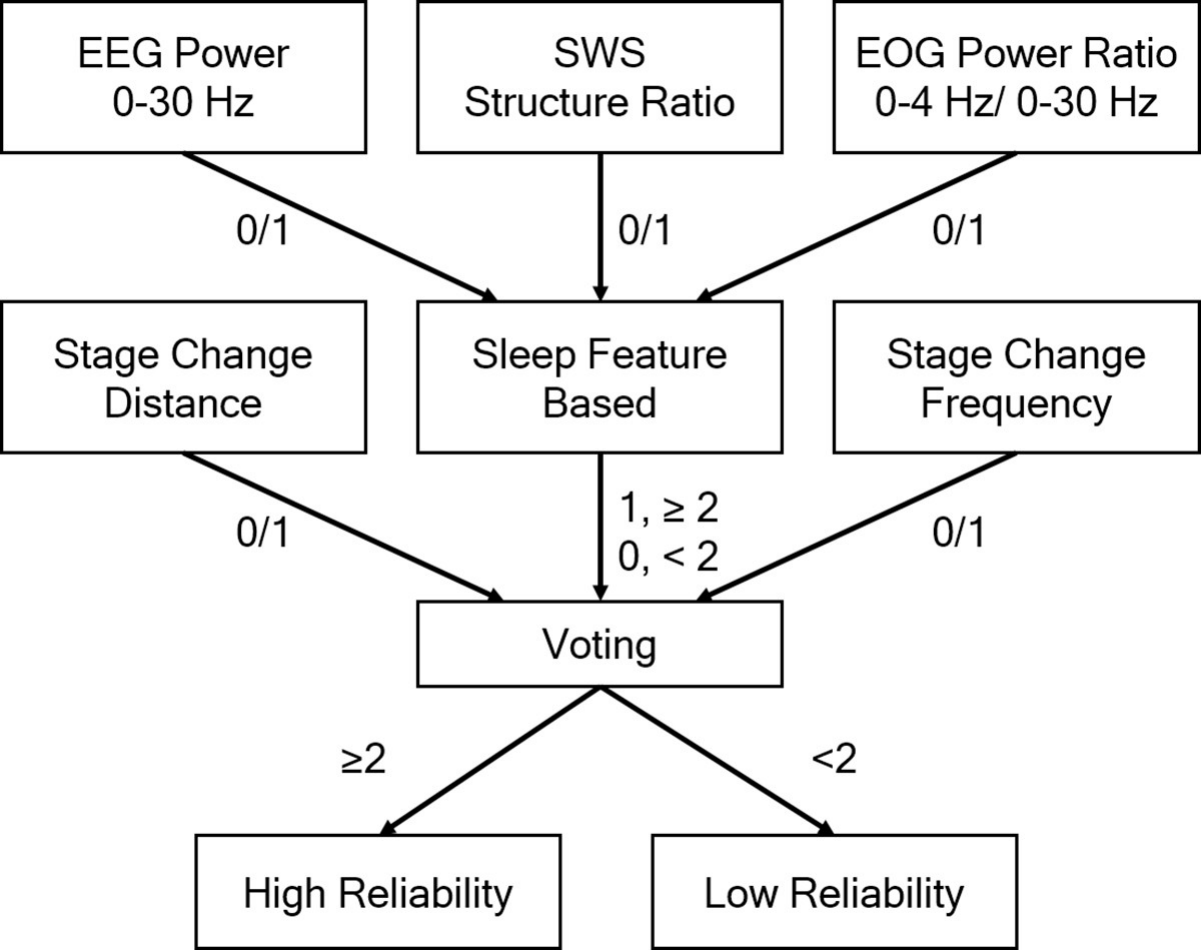
The architecture of the voting process. According to the value of the SWR, SCD, and SCF features, the voting process determines a scored epoch as a high reliability or low reliability.

To enhance the reliability analysis to detect epochs with low reliability, we first calculated the SWR, SCD and SCF features. Next, a voting process was applied, as shown in Fig 7, to determine the level of reliability for each epoch. Finally, an expert only needs to rescore epochs with a low level of reliability.

As presented in Session 2.1, the three SWR features, 0–30 E, SWS E, and 0–4 O, were calculated. If one SWR feature is within the boundary ranges, the output of the SWR feature is set to 0 representing a low reliability. Otherwise, this SWR feature is set to 1 representing a high reliability. If two or more SWR features are 1 (≤2), the vote for the overall SWR feature is set to 1 (high-reliability). Otherwise, the vote is 0.

According to Sessions 2.2 and 2.3, the vote of SCD is 1 (high-reliability) if the SCD value of an epoch is higher than 6. Otherwise, the vote of SCD is 0. Similarly, the vote of SCF is 1 if the SCF value of an epoch is equal to or less than 2. Otherwise, the vote of SCD is 0.

Finally, we sum the total value of the votes. If the number of total votes is higher than 1 (≤2), the epoch is identified as high reliability. Otherwise, the epoch is identified as low-reliability and an expert should rescore the epoch.

## Experimental results

Two sleep experts, scorer 1 and scorer 2, participated in our experiments to test the performance of our system. Each scorer scored the 30 subjects’ PSG data with (HCSS group) and without the assistance of the HCSS system (Manual Group) separately. The PSG data was given to the scorers in a random order and the scoring agreement was assessed between the fully manual scoring and the scoring collaborated with the HCSS for each scorer. The manual scoring time spent on fully manual scoring and HCSS scoring was also evaluated and compared in the experiment.

**Fig 8:**
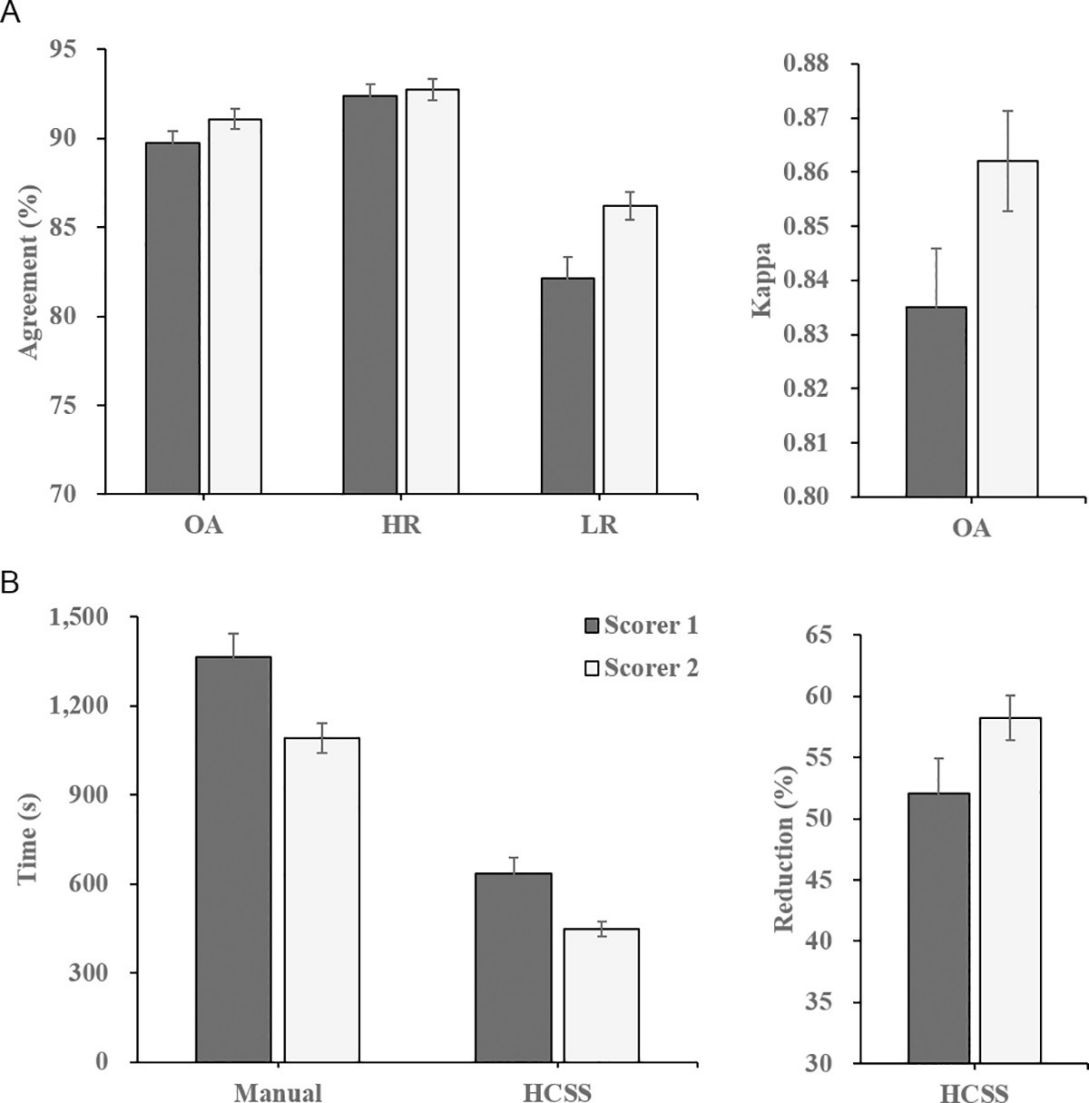
Evaluation of the HCSS system. (A) The agreement in overall, high-reliability and low-reliability epochs, along with the kappa coefficient between the manual scorings and the HCSS system collaborated scorings. (B) The average of the scoring time for one subject spent in manual and HCSS groups. Percentage of reduced manual scoring time with the assistance of the HCSS system; OA: overall, HR: high-reliability, LR: low-reliability.

Fig 8A shows the agreements between fully manual and HCSS scorings for scorer 1 and scorer 2, including overall epochs, epochs of high-reliability and epochs of low reliability. The overall agreement for scorer 1 and scorer 2 are 89.74% and 91.1%, respectively. The average agreements are 92.37% and 92.77% in the high-reliability epochs and are 82.12% and 86.23% in the low reliability epochs, respectively. The kappa coefficients [34] between each scorer’s manual scorings and the results collaborated with the HCSS system are 0.84 and 0.86, respectively. This indicates excellent agreement (>0.8). These experimental results show that a human-computer collaborative sleep scoring method can perform well during stage transitions. Fig 8B shows the cost in time for manual scoring with and without the assistance of the HCSS system. Since the required scoring time is different for PSG data with different sleep quality, the percent reduction in manual scoring time with the assistance of the HCSS compared with fully manual scoring for the two scorers is also presented. The average manual scoring time spent on an overnight PSG data for scorers 1 and 2 without the assistance of HCSS system are1360 and 1090 seconds, respectively. The manual scoring time using the HCSS system are reduced to 636 and 446 seconds for scorer 1 and scorer 2, respectively. The scorer needed approximately 0.5 seconds and 1 second to identify epochs with high-reliability and low reliability, respectively. The experimental results show that the proposed HCSS system can enable an approximately 55.12% reduction in manual scoring time (52.03% for scorer 1 and 58.21% for scorer 2).

A 10-fold cross-validation was used to compute the misclassification error for the reliability analysis of 30 PSG data. The misclassification error was 0.2824 and 0.276 for scorer 1 and scorer 2 using our method.

Table 2 shows the agreement of scoring between the scorers and automatic scoring or HCSS collaborative scoring with respect to the patients with good and poor sleep efficiency (SE). SE higher than or equal to 85% is considered good sleep quality and SE lower than 85% is considered poor sleep quality. The agreement of fully automatic sleep scoring decrease 5% when SE is lower than 85%, however, the agreement can be higher than 87.27% for data characterized as low sleep efficiency when the HCSS system is adopted. The results suggest that cooperative scoring between humans and machines is superior to fully automatic scoring systems.

**Table 2:** Agreement comparison with respect to data with good and poor sleep efficiency.

	Agreement		
Sleep Efficiency	Gold Standard Vs. Automatic	Scorer 1 Vs. HCSS	Scorer 2 Vs. HCSS
≥85%	84.74%	88.88%	89.53%
< 85%	79.02%	88.44%	87.27%

Table 3 shows the comparison of agreement of the gold standard, scorer 1 and scorer 2 in terms of manual scoring and using our method. Compared with the gold standard, the agreement of scorer 1 and scorer 2 are both higher than 85%, improving approximately 1% when using our method. Compared to a purely automatic method [5] with the same PSG data as shown in Table 2, our method has a higher agreement in both scorers.

**Table 3:** Comparison of agreement between the gold standard, scorer 1 and scorer 2 for manual scoring and the HCSS method.

Agreement	Epoch	Scorer 1	Scorer 1+HCSS	Scorer 2	Scorer 2+HCSS
**Gold Standard**	OA	85.76%	86.46%	85.99%	86.97%
	HR	89.96%	90.62%	90.21%	91.29%
	LR	74.02%	74.47%	74.07%	74.67%
**Scorer 1**	OA		91.41%	86.83%	87.83%
	HR		93.08%	90.21%	91.60%
	LR		86.60%	77.30%	76.81%
**Scorer 1+HCSS**	OA			87.93%	92.09%
	HR			91.20%	95.67%
	LR			78.68%	81.70%
**Scorer 2**	OA				89.74%
	HR				92.38%
	LR				82.12%

OA: overall; HR: high-reliability; LR: low-reliability.

## Discussion

In this paper, a human-computer collaborative sleep scoring system is proposed. A rule-based automatic sleep scoring method is utilized to perform the first-run scoring and the level of reliability for each epoch is also analyzed based on the sleep features and the stage change observations. The percentage of low-reliability epochs where sleep experts must rescores is shown in Table 4.

**Table 4:** Percentage of low-reliability epochs across sleep stages where sleep experts have to examine.

Subject No.	LR (%)	Subject No.	LR (%)	Subject No.	LR (%)
1	24.36%	11	25.69%	21	27.11%
2	31.07%	12	30.71%	22	29.48%
3	18.24%	13	31.20%	23	17.81%
4	25.96%	14	14.78%	24	39.17%
5	25.43%	15	20.10%	25	24.22%
6	30.98%	16	15.54%	26	16.50%
7	25.95%	17	23.93%	27	40.30%
8	26.12%	18	28.72%	28	38.08%
9	25.89%	19	46.09%	29	25.91%
10	27.73%	20	23.24%	30	23.70%

Low Reliability Epoch Percentage: 26.8%±1.33% S.E.M.

The average overall agreement, kappa coefficient, and percentage of scoring time reduced between the complete manual scorings and our HCSS system are 88.47%, 0.825, and 55.28%, respectively. Table 5 shows a comparison of agreement between the different automatic scoring methods [5, 35]. By using our method, sleep experts only need to score 26.8% of the epochs that are labeled as low-reliability and the agreement of gold standard both in good sleepers and bad sleepers can reach 86.97%.

**Table 5:** Comparison of agreement between different automatic scoring methods.

Method	Private Database	Gold Standard Agreement
Rule-Based [5]	16 Good Sleepers	85.85%
GA-fuzzy [35]	16 Good Sleepers	87.93%
HCSS	15 Good+15 Bad Sleepers	86.97%

A good sleeper’s sleep efficiency is equal to or higher than 85%, and a bad sleeper’s sleep efficiency is lower than 85%.

**Fig 9:**
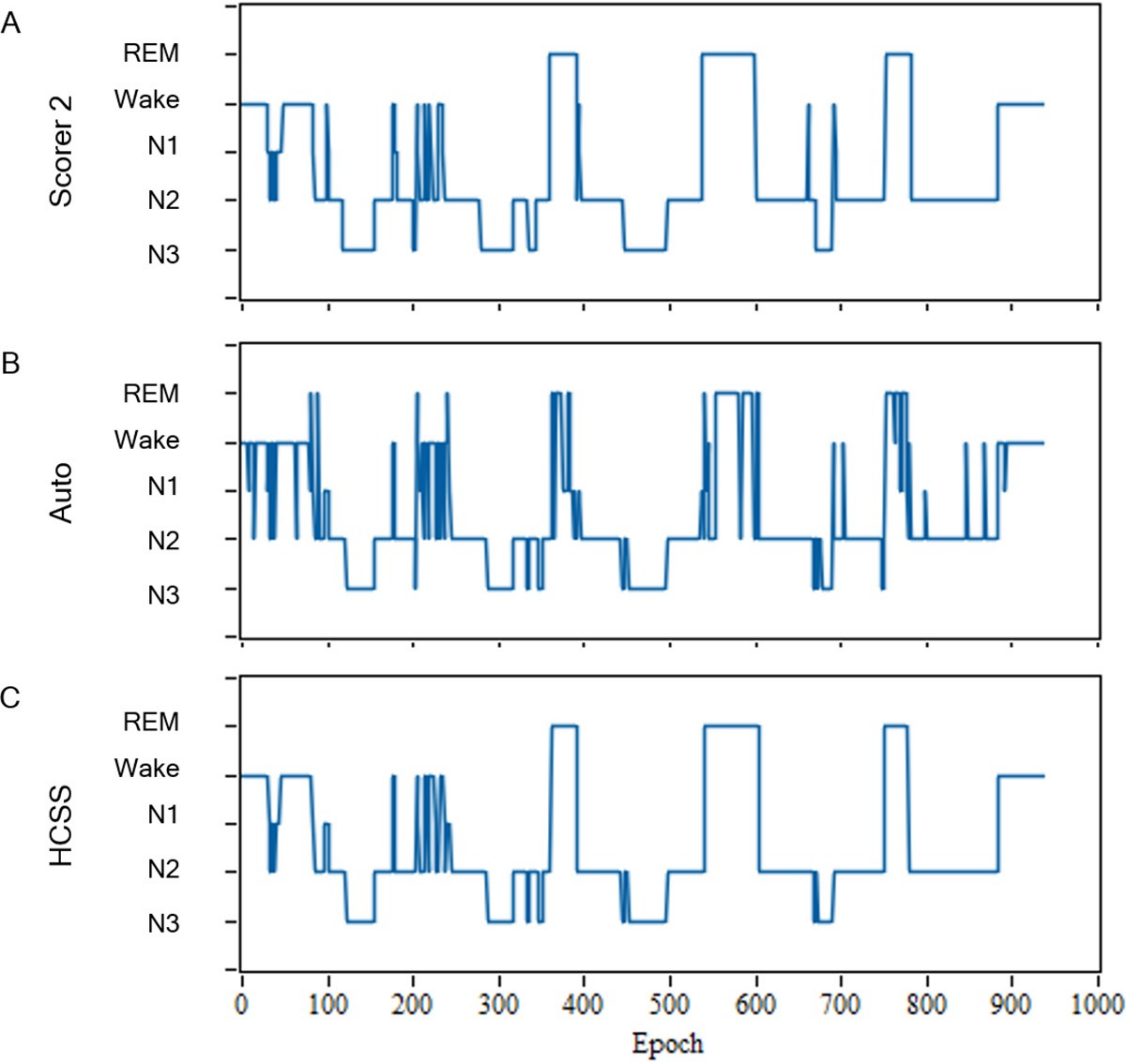
Hypnograms of the subject no. 4. The hypnograms scored by fully manual scoring (scorer 2) (A), fully automatic staging (B) and the HCSS system (C).

Similar to the AASM interscorer program report, most of the scoring disagreements occur during stage transitions where sleep stage changes frequently within a short period of time. Therefore, stage change frequency (SCF) and stage change distance (SCD) are proposed in this paper to be used to estimate the reliability level of scoring in each epoch. The epochs would be appropriately assigned to an expert or a computer to score according to the reliability analysis. The experimental results demonstrate that the proposed strategy can tremendously reduce manual scoring time while maintaining high scoring quality. Fig 9 shows the hypnograms of subject no. 4 (SE: 82.98%), including the manual scoring by the scorer, the 1st stage fully automatic staging, and the proposed HCSS system. It was observed that the hypnogram of the HCSS system is closer to the hypnogram scored by the expert in the stage transitions compared with the fully automatic staging.

For flexibility, we propose two operational modes for the reliability analysis as summarized in Table 6. An epoch with SCD > 5 or SCF < 3 is identified as high-reliability under the normal mode (level 1). The less stringent mode (level 2) identifies more epochs as high-reliability by loosening the criteria, using SCD > 4 or SCF < 4. Reliability analysis for overnight PSG recordings from 30 subjects shows that 73.21% and 82.07% of the epochs were flagged as high-reliability for automatic staging by level 1 and level 2, respectively. The agreements were 88.66% and 86.22% under the normal and less stringent mode, respectively. These agreements are higher than the interscore standard (82.6%) from AASM’s reliability program [16] and suggest that the rule-based automatic sleep scoring method perform well in scoring high-reliability epochs. The scorers can choose different operation modes depending on how much they trust the HCSS system or how many low-reliability epochs they are willing to rescore.

**Table 6:** Agreement comparisons with respect to different threshold levels in the reliability analysis.

**Level 1: SCD > 5, SCF < 3** **High reliability (%)** 73.21%	**Agreement** 88.66%	**Level 2: SCD > 4, SCF < 4** **High reliability (%)** 82.07%	**Agreement** 86.22%

## Conclusion

Automatic sleep staging methods based on multi-channel signals and single-channel recordings as well as various deep neural networks such as DBNs, CNNs, and LSTM have been developed to efficiently reduce the amount of time and effort for sleep scoring. However, the methods employed are not often published. Thus, the unexplained results from an uninterpretable black box limit the applicability of these methods in clinical diagnosis. Instead of fully automatic or fully manual scoring, this paper proposes a human-computer collaborative approach for sleep analysis. The experimental results demonstrate feasibility, and the proposed approach can also be included with other various methods.

In the future, more PSG data with various sleep quality and more sleep scorers will be included in experiments to evaluate the applicability of the HCSS system. A user-friendly interface with complete assistant functions will also be designed to provide a high level of usability for the scorers to achieve efficient sleep diagnosis.
